# Graded Mirror Self-Recognition by Clark’s Nutcrackers

**DOI:** 10.1038/srep36459

**Published:** 2016-11-04

**Authors:** Dawson Clary, Debbie M. Kelly

**Affiliations:** 1 Department of Psychology, University of Manitoba, Winnipeg, Manitoba, CANADA

## Abstract

The traditional ‘mark test’ has shown some large-brained species are capable of mirror self-recognition. During this test a mark is inconspicuously placed on an animal’s body where it can only be seen with the aid of a mirror. If the animal increases the number of actions directed to the mark region when presented with a mirror, the animal is presumed to have recognized the mirror image as its reflection. However, the pass/fail nature of the mark test presupposes self-recognition exists in entirety or not at all. We developed a novel mirror-recognition task, to supplement the mark test, which revealed gradation in the self-recognition of Clark’s nutcrackers, a large-brained corvid. To do so, nutcrackers cached food alone, observed by another nutcracker, or with a regular or blurry mirror. The nutcrackers suppressed caching with a regular mirror, a behavioural response to prevent cache theft by conspecifics, but did not suppress caching with a blurry mirror. Likewise, during the mark test, most nutcrackers made more self-directed actions to the mark with a blurry mirror than a regular mirror. Both results suggest self-recognition was more readily achieved with the blurry mirror and that self-recognition may be more broadly present among animals than currently thought.

Since its development^1^, the mark test has incited controversy over whether non-human animals are capable of recognizing themselves in a mirror and whether this ability supports a concept of general self-awareness. Although advocated by some^2^, such rich interpretations involving self-awareness are not necessary. Instead, a more moderate interpretation is generally accepted that the mark test indicates the ability to form a mental representation of one’s body or appearance, though this need not necessitate other traits implicated with self-awareness (e.g., Theory of Mind, introspection, awareness of death)^3^. To implement the mark test, an animal is typically provided with exposure to a mirror, allowing the subject to learn that the image in the mirror corresponds both with their appearance and movements. During the crucial test, an animal is inconspicuously marked, such that it cannot see the mark without using a mirror. If the animal increases the number of attempts to inspect or remove the mark from its own body (and not the reflection) in the presence of a mirror, relative to behaviours directed to this area of the body when it was unmarked, it suggests the animal has used the mirror to infer that its appearance has been altered and, thus, necessary to correct. Although the mark test has strength in its simplicity, the results from using this approach are not always easily interpretable. For instance, if the animal does not make any attempts to remove the mark, perhaps owing to species’ differences in motivation to interact with mirrors or to remove marks placed on their bodies^3^,^4^, the interpretation of an absence of evidence is problematic. Therefore, the development of supplemental tasks assessing self-recognition, as well as variations on the mark test, are needed to provide more in-depth understanding of mirror self-recognition in non-human animals.

So far, evidence has accumulated that great apes are capable of passing the mark test^5^,^6^, as well as more recent research suggesting other large-brained mammals, such as dolphins^7^ and elephants^8^, may also share this ability. Corvids are a family of large-brained birds recognized for their impressive cognitive abilities, feats that often challenge the notion of unique human or primate cognition^9^,^10^,^11^,^12^. Studying the caching behaviour of corvids, in which birds hide food during times of plenty for later retrieval when food is scarce, has revealed many cognitive abilities not previously known^13^. In particular, observing interactions between birds caching food and birds trying to steal those caches has highlighted corvids’ prowess for social cognition. Indeed, competition for caches likely fuelled an evolutionary arms race between caching and thieving birds, scaffolding the cognitive abilities of corvids^14^. For example, caching corvids change their strategies for hiding food depending on whether they are observed^14^, the identity and perspective of the observer^15^,^16^,^17^,^18^, and their own experience as thieves^11^,^19^. Clark’s nutcrackers, for instance, prevent theft by suppressing caching in the presence of conspecific observers^19^ – a finding we capitalized on to study mirror self-recognition.

Evidence of mirror self-recognition in corvids, as expressed in the mark test, is mixed: European magpies passed the mark test^9^; however, a study using jackdaws found no evidence of self-recognition and instead suggested tactile cues provided by the marks, rather than mirror use, best explained the findings from both jackdaws and magpies^20^. Given the success of caching paradigms in revealing the cognitive abilities of corvids, we applied this approach to the study of mirror self-recognition. Because nutcrackers have shown a social suppression of caching in our previous research^19^, we explored whether a similar reluctance to cache would occur with a mirror. That is, would the birds view their mirror image as a potential thief and suppress caching, or would they recognize the reflection as themselves and cache freely, as if alone? Thus, we allowed ten mirror-naïve nutcrackers to cache alone, with another nutcracker, and with a regular or blurry mirror over multiple trials (Fig. 1). When viewing a mirror, two sources of information are present: identity information gained from fine-scale details, and contingent motion information gained from the correspondence between one’s own movements and that of the reflection^21^. By applying a film of faux window frosting to a regular mirror to create a blurry mirror, fine details were removed from the reflected image. Therefore, birds could use the contingent motion information provided by the blurry mirror, but not clear identity information. The blurry mirror allowed us to evaluate the roles of identity and contingent motion information: Would removal of identity information disrupt self-recognition through loss of an informative cue or facilitate self-recognition by eliminating cue competition with contingent motion? Removing identity information may evoke uncertainty about the identity of the individual that is being reflected, and thus may interfere with the ability to self-recognize. However, this might be an anthropomorphic interpretation, as clear identity information is most often obtained when viewing other individuals, as rarely does a bird in its natural setting have an opportunity to obtain a clear reflection of itself. In contrast, contingent motion is frequently associated with oneself and rarely occurs in the presence of other individuals. Therefore, the associative learning history of individuals may introduce potential for cue competition between identity and contingent motion information, whereby removing identity information aids one’s ability to determine the reflection is oneself. Our study attempts to disentangle these alternatives by using regular and blurry mirrors, thereby manipulating the availability of clear identity information, to assess how identity and contingent motion information contribute to self-recognition.

Overall in our study, birds were given 45 minutes to eat or cache 50 pine nuts in ice-cube trays filled with sand. Trays were positioned within each compartment of a two-compartment cage, so the adjacent cage compartment ‘mirrored’ the focal bird’s compartment. After the caching trials, we conducted the mark test, again incorporating the blurry mirror to further evaluate the roles of identity and contingent motion information.

## Methods

### Subjects

Seventeen Clark’s nutcrackers (*Nucifraga columbiana*) participated in the experiment: ten focal birds (five female; five male) and seven observers (three male; four female). Sample size was based on previous studies to ensure adequate detection power^10^,^11^,^19^. All focal birds participated in each experiment described. All birds had previous experience in caching experiments, but had no laboratory experience with mirrors or being marked. Birds were housed in individual cages (73 × 48 × 48 cm) and fed *ad libitum* on non-experimental days. Observer birds were fed *ad libitum* at all times. Diet consisted of turkey starter, parrot pellets, sunflower seeds, peanuts, pine nuts, mealworms, oyster shells, and a vitamin powder supplement. The colony room was maintained at 21 °C and a 12:12 day:night cycle with light onset at 0700. Birds had been housed in the laboratory for 10–16 years. All procedures were approved by the University of Manitoba’s Animal Care Committee (protocol #F10-029) and in compliance with the standards of the Canadian Council on Animal Care.

### Apparatus

Birds cached in plastic ice cube trays filled with sand (26 wells arranged in a 13 × 2 matrix; 49.5 × 11.5 cm), placed into each side of a two-compartment cage (overall dimensions: 123 × 63.5 × 66.5 cm) in an isolated experimental room. A wooden dowel perch was placed in the corner of each compartment. Trays were arranged along the cage wall nearest to the adjacent compartment. The two-compartment cage (and trays) could be separated by a removable clear acrylic or mirror partition (65 × 62 cm). The cage was placed on a table surrounded by white curtains to create a uniform viewing environment. All trials were recorded using an EverFocus^®^ 1/3′′ color digital camera and the motion tracking program, BiObserve.

### General procedures

Focal birds were food deprived 24 hours prior to participating in a trial (one trial per week). Each trial consisted of a *Caching Session* and a *Retrieve Session*. On the first day, a bird was placed in one side of the two-compartment cage and allowed to eat and cache 50 pine nuts (i.e., *Caching Session*) for 45 minutes. The birds were then returned to the colony room and supplemented with a restricted amount of regular feed to maintain their weight after food deprivation. On the next day, the birds were returned to the experimental cage to recover their caches for 45 minutes (i.e., *Retrieve Session*). If caches remained in the tray or cage after the session, additional sessions were conducted until all caches were recovered. This was done to prevent memories of previous caches from contaminating the birds’ caching decisions on future trials. Birds were returned to an *ad libitum* diet after completing the *Retrieve Session*.

### Experiment 1

#### Caching with a blurry mirror

The birds participated in one habituation trial, conducted to prevent the novelty of the environment from affecting subsequent trials, followed by six Baseline trials before experiencing test conditions. Both the habituation trial and Baseline trials followed the same procedures (see below). After Baseline trials, each bird was given six blocks of test trials, each block consisting of one Alone, Observed, Mirror, and Blurry Mirror trial, conducted in randomized order. For all conditions, the number of pine nuts cached (both in the tray and external) and eaten during the *Caching* and *Retrieve Sessions* was documented.

##### Baseline/Alone

During Baseline (and the one habituation trial), a clear acrylic partition separated the two compartments of the cage. The focal bird was placed in one compartment with a caching tray placed along the clear partition. The other compartment contained an identical caching tray placed along the clear partition so that it was parallel with the other tray. This condition assessed each bird’s normal caching when alone and without cache loss. Alone trials were identical to Baseline, but conducted concurrently with the other test conditions.

##### Mirror/Blurry Mirror

During this condition, either a regular mirror or a blurry mirror was inserted behind the clear acrylic partition. The blurry mirror was created by covering a mirror with a film of faux window frosting (Artscape Inc. Texture Twelve), such that it obscured fine details, but remained reflective. Once the bird was returned to the colony room after the *Caching Session,* the experimenter artificially pilfered half of the caches made by the focal bird to simulate cache theft, as would occur if another bird had witnessed the caching event^19^. This manipulation created a situation where the birds should suppress caching if they interpret the mirror image as another bird, whereas they should not suppress caching if they interpret the mirror image as their own reflection.

##### Observed

An observer bird was placed in the compartment adjacent to the focal bird. Each focal bird was paired with the same observer for the duration of the experiment. All observers were of the same sex and approximate size as the focal bird. Observers were provided with pine nuts to eat and cache, so the focal bird would be exposed to behaviours they would see during the mirror conditions. The focal bird was returned to the colony room first, followed by the observer ten minutes later. This procedure was implemented to create a period of uncertainty for the focal bird, during which the observer was left alone with the caching trays, though in reality did not have access to the focal bird’s tray. As with the mirror conditions, the experimenter artificially pilfered half of the focal bird’s caches prior to the *Retrieve Session*.

### Experiment 2

#### Caching with a half mirror

Experiment 2 occurred immediately following Experiment 1. General procedures were identical to Experiment 1, except each bird was given three blocks of test trials, each block consisting of one Alone, Observed, and Half Mirror trial, presented in randomized order. The half mirror (37 × 62 cm) was placed behind the clear acrylic partition so that it covered the left half of the opening between cage compartments. One bird (Reorx) was paired with a new observer after the second Observed trial due to the unavailability of the original observer.

### Experiment 3

#### Caching after devoted mirror sessions

Experiment 3 occurred immediately following Experiment 2. General procedures were identical to Experiment 1, except each bird was given three blocks of test trials, each block consisting of one Alone, Observed, and (Full) Mirror trial, presented in randomized order. Additionally, birds were given exposure sessions, lasting 20 minutes each, during which the birds were individually placed in the experimental cage with just the half mirror, no caching trays, and the clear partition only extended to the edge of the half mirror. Thus, the birds had access to the back of the mirror by entering the adjacent cage compartment. These sessions were conducted on weekdays the birds were not scheduled to participate in either a *Caching* or *Retrieve Session*. Birds received 27–30 of these sessions for a total of 9–10 hours of half mirror exposure in addition to the exposure gained during caching trials with a full mirror. As caching trials were conducted over two day periods, either Tuesday/Wednesday or Thursday/Friday, the birds were always given a devoted half mirror exposure session on the day immediately prior to their participation in caching trials.

### Experiment 4

#### Mark test

**Figure 2:**
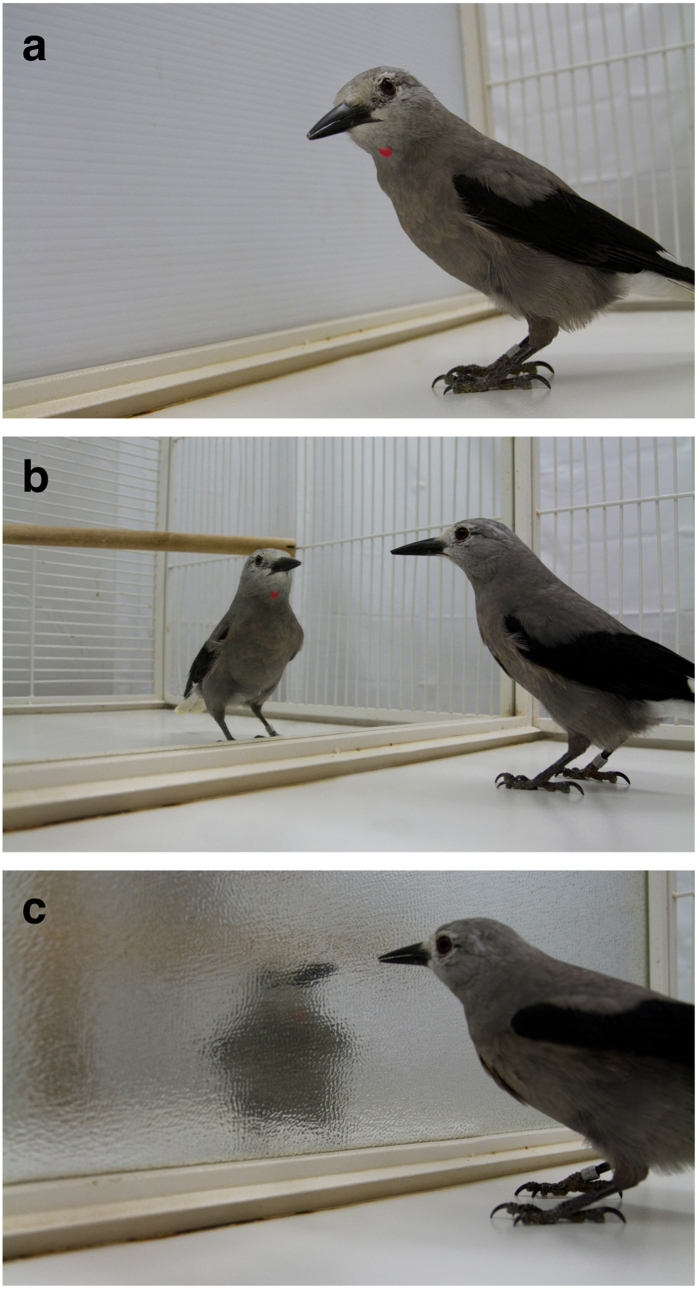
Photos showing the (**a**) barrier, (**b**) regular mirror, and (**c**) blurry mirror conditions with the red mark.

Experiment 4 occurred immediately following Experiment 3. Trials were conducted in the same environment as the previous three experiments. All birds experienced two 20-minute trials each day during which a bird was marked with a red, grey, or sham mark^9^,^20^. [Marks were made from coloured adhesive dots (diameter = 6 mm). Grey marks were made to match the nutcracker’s plumage by painting them with Ominous Grey (50YR 26/023) CIL^®^ paint. Red marks weighed on average 6325 ± 969 μg; grey marks weighed on average 7850 ± 814 μg (mean ± standard deviation)]. Before a trial, the experimenters covered the bird’s head so a mark could be pressed onto the bird’s throat feathers outside the bird’s normal range of sight (Fig. S1). The experimenter then gently pressed on various parts of the bird’s body so tactile cues were not exclusively to the mark region. In the case of the sham mark, a mark was pressed onto the bird as described, but immediately removed before pressing other parts of the body. Next, the bird was placed into the cage, where an opaque white barrier, a regular mirror, or a blurry mirror separated the cage compartments (Fig. 2). Birds experienced 18 trials: two trials with each mark type (i.e., red, grey, no mark) were conducted for each of the three viewing conditions (i.e., barrier, blurry mirror, regular mirror), presented in pseudo-randomized order so birds experienced each condition-mark combination once before repeating any trial type. The first trial type each bird experienced was roughly counterbalanced (9 condition-mark combinations and 10 birds).

### Statistical Analysis

For the caching experiments (Experiments 1–3), number of caches was documented after each session. For the mark test (Experiment 4), the number of actions (with either a foot or the beak) a bird made to the mark region, to any other part of the plumage, and any feather ruffling (either by shaking the head or shoulders) that could be used to detect disturbances of the plumage was scored. A conservative approach was used to analyze actions during the mark test. The absolute number of actions rather than proportion of total actions in a trial were examined. This approach was used because identifying the mark, at times, stimulated further preening, reducing the proportion of mark actions during trials in which the birds identified the mark. Furthermore, there were large between individual differences, with some birds engaging in very few or no self-directed actions, which made analyses based on proportions unfeasible. However, we report all actions in Table 1 for reference. All trials were scored by DC, and a second rater scored a subset of 25 trials as an inter-rater reliability check. The scoring of these behaviours was highly correlated between the two raters (*r* = 0.97). On items of disagreement the two scorers came to a consensus on the correct decision to be used in the final analysis.

Linear mixed-effects models were constructed inputting mirror condition and mark condition (for the mark test) as fixed effects, and subject inputted as a random effect to account for repeated measures taken on each bird. Residual plots indicated assumptions of normality were valid. No birds were excluded from the analyses, and all birds completed all conditions for a balanced design with similar variances between groups. Analyses were conducted in R version 3.1.2 using the *lme4*^22^ and *multcomp*^23^ packages.

## Results

### Experiments 1

#### Caching with a blurry mirror

**Figure 3:**
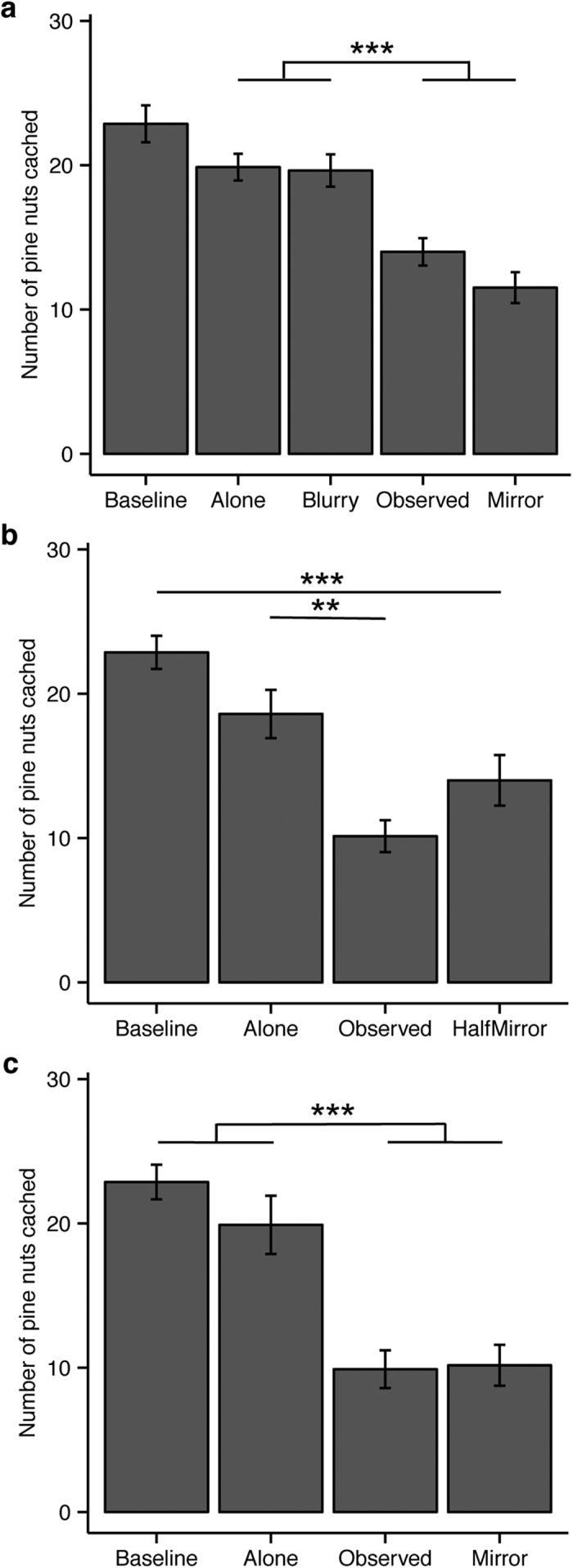
Mean number of pine nuts cached per trial (±SEM) by the birds (*n* = 10) during each caching experiment. (**a**) During Experiment 1 the birds cached alone, in the presence of an conspecific, with a regular mirror, and with a blurry mirror. (**b**) During Experiment 2 the birds cached alone, in the presence of a conspecific, and with a half mirror that covered one vertical half of the cage. (**c**) During Experiment 3 the birds cached alone, in the presence of a conspecific, and with the full sized mirror. Trials were run concurrently with devoted mirror exposure sessions provided throughout the week. **p < 0.01 ***p < 0.001.

There was an overall effect of caching condition (*F*_(*4,286*)_ = 22.741, *p* < 0.001). Relative to caching during the Alone condition (*M* ± *SEM* = 19.87 ± 0.93), the nutcrackers suppressed caching when observed by another bird (*M* ± *SEM* = 14.00 ± 0.95, *z* = 4.242, *p* < 0.001) and with the regular mirror (*M* ± *SEM* = 11.52 ± 1.07, *z* = 6.038, *p* < 0.001; Fig. 3a), indicating the nutcrackers perceived a risk to their caches during these conditions. However, the birds did not suppress caching with the blurry mirror (*M* ± *SEM* = 19.63 ± 1.12) relative to when caching alone (*M* ± *SEM* = 19.87 ± 0.93, *z* = 0.169, *p* = 1.000), suggesting the birds did not interpret the blurred image as a potential thief. To understand the discrepancy between the regular and blurry mirror conditions we further investigated two questions: (1) could the birds learn to self-recognize with a regular mirror, and (2) did the birds self-recognize with the blurry mirror?

### Experiments 2

#### Caching with a half mirror

To examine whether giving the birds an opportunity to learn the properties of the mirror would allow self-recognition to manifest with the regular mirror, we conducted additional trials using a half mirror covering one vertical side of the testing compartment. The half mirror allowed birds to examine the appearance and disappearance of their reflection at the mirror’s edge, though a clear acrylic partition prevented access behind the mirror. Despite this experience, the main effect of caching condition remained (*F*_(*3, 137*)_ = 21.082, *p* < 0.001). The nutcrackers continued to suppress their caching with the half mirror (*M* ± *SEM* = 14.00 ± 1.75) relative to the Alone (*M* ± *SEM* = 18.60 ± 1.67, *z* = 2.312, *p* = 0.069) and Baseline conditions (*M* ± *SEM* = 22.87 ± 1.15, *z* = 5.147, *p* < 0.001; Fig. 3b), and preferred to cache on the non-mirror side of the tray (Fig. S2).

### Experiments 3

#### Caching after devoted mirror sessions

We provided the birds with devoted exposure sessions to mirrors (i.e., no caching trays or clear acrylic partition) on non-test days, during which the birds could explore the adjacent compartment behind the mirror. Even with these sessions the main effect of caching condition remained (*F*_(*3,137*)_ = 26.816, *p* < 0.001), as cache suppression persisted with the regular mirror (*M* ± *SEM* = 10.17 ± 1.42) relative to caching alone (*M* ± *SEM* = 19.90 ± 2.02, *z* = 4.676, *p* < 0.001; Fig. 3c, Fig. S3). Thus, when uncertainty about the identity of the mirror image was paired with the risk of cache theft, the birds were unable to learn to self-recognize with the regular mirror.

### Experiments 4

#### Mark test

**Figure 4:**
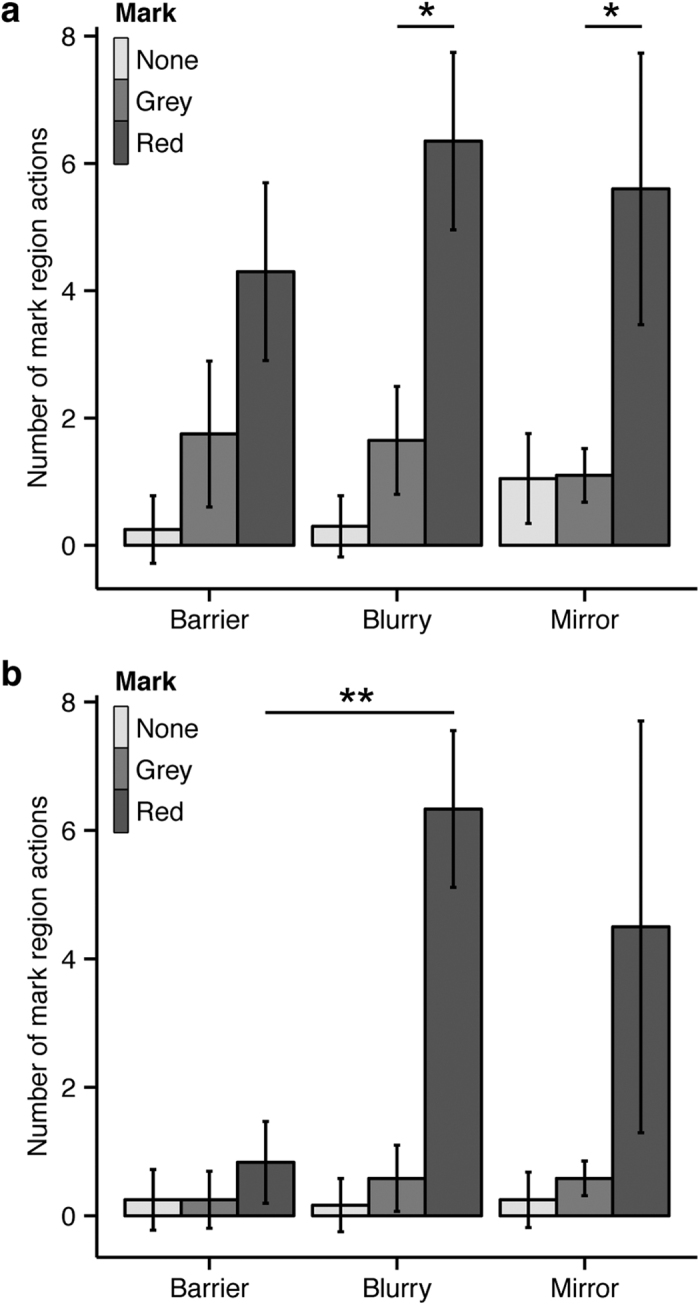
Mean number of actions made to the mark region per trial (±SEM) for (**a**) all ten birds and (**b**) the subset of six birds that showed evidence for mirror use. *p < 0.05 **p < 0.01.

There was an overall effect of mark colour as the birds made more actions to the mark-region with the red test mark than with the grey control mark (*F*_(*2,162*)_ = 16.513, *p* < 0.001), but only with the regular (*M*_*Red*_ ± *SEM* = 5.60 ± 2.13, *M*_*Grey*_ ± *SEM* = 1.10 ± 0.42, *z* = 2.887, *p* < 0.026) and blurry (*M*_*Red*_ ± *SEM* = 6.35 ± 1.39, *M*_*Grey*_ ± *SEM* = 1.65 ± 0.85, *z* = 3.015, *p* = 0.018) mirrors and not with the opaque barrier (*M*_*Red*_ ± *SEM* = 4.30 ± 1.40, *M*_*Grey*_ ± *SEM* = 1.75 ± 1.14, *z* = 1.636, *p* = 0.437; Fig. 4a). There was no main effect of condition (*F*_(*2, 162*)_ = 0.293, *p* = 0.747), nor a condition by mark interaction (*F*_(*4,162*)_ = 0.429, *p* = 0.787) for mark-region directed actions. Upon visual inspection of each bird’s performance (Table 1), it became evident there were two groups of birds with differing patterns. For additional analyses, we divided the birds into two groups. One group of birds (*Mirror advantage, n* = 6) showed an advantage for making mark-region actions in the presence of one or both of the mirror types. The second group (*Non-mirror visual strategy, n* = 4) showed elevated actions to the mark-region with the red mark (but not grey mark) during the control condition with the opaque barrier. Isolating certain groups of animals or analyzing specific individuals is common for mirror recognition studies^7^,^8^,^9^,^24^, as doing so can provide evidence that self-recognition is within the theoretical capabilities of a species, though it may not be a ubiquitous feature within all individuals of the species (at least in the context of the specific test administered). For the *Mirror advantage* group we included only birds that made *at least* 50% more mark-region actions with the red mark during mirror conditions than *any* control condition (i.e., all Barrier conditions and Mirror conditions with grey and sham mark procedures). This stringent criterion was chosen to ensure only the birds with the clearest evidence of possible mirror use (scaled to each bird’s own propensity for self-directed actions) were included in the *Mirror advantage* group, and therefore this conservative criterion excluded a bird such as Krusty (see Table 1), who also showed higher mark-region actions in some mirror conditions, but also had elevated mark-region actions during control conditions.

The *Mirror advantage* group showed a main effect of mark colour (*F*_(*2, 94*)_ = 9.115, *p* < 0.001), no effect of condition (*F*_(*2,94*)_ = 2.097, *p* = 0.129), and no mark by condition interaction (*F*_(*4, 94*)_ = 1.805, *p* = 0.134). Planned contrasts revealed this subset of birds made more actions to the red mark with the blurry mirror (*M* ± *SEM* = 6.33 ± 1.22) than with the opaque barrier (*M* ± *SEM* = 0.83 ± 0.64, *z* = 3.309, *p* = 0.007), which was not statistically different from the actions directed toward the grey mark during the Barrier condition (*M* ± *SEM* = 0.25 ± 0.44, *z* = 0.351, *p* = 1.000). One bird (Fido) also used the regular mirror to reliably identify the red test mark, as shown by more mark-region actions occurring with the regular mirror than during control conditions (Fig. 4b, Table 1). There was no effect of trial on the number of mark-region actions when this factor was included in the model (*F*_(*1, 85*)_ = 0.091, *p* = 0.764), nor a mark by condition by trial interaction (*F*_(*4, 85*)_ = 0.929, *p* = 0.451). Importantly, we found no evidence these six birds used tactile cues to detect the mark, as the birds did not ruffle their feathers more (a common avian response to remove debris from plumage) when marks were applied (*F*_(*2, 94*)_ = 0.365, *p* = 0.695; Fig. S4). Instead, these six birds used the mirror spontaneously, without explicit training^25^, to detect the mark.

The *Non-mirror visual strategy* group showed a main effect of mark colour (*F*_(*2,60*)_ = 8.114, *p* = 0.001), but did not show a statistical difference for the number of mark-region actions to the red mark between the barrier condition (*M* ± *SEM* = 9.50 ± 2.91) and the regular (*M* ± *SEM* = 7.25 ± 2.58, *z* = 0.761, *p* = 0.965) or blurry mirror (*M* ± *SEM* = 6.38 ± 3.02, *z* = 1.057, *p* = 0.841) conditions. The number of mark-region actions was greater during the Barrier condition with the red mark (*M* ± *SEM* = 9.50 ± 2.91) than with no mark (*M* ± *SEM* = 0.25 ± 0.91, *z* = 3.128, *p* = 0.013). There was no effect of condition (*F*_(*2,60*)_ = 0.259, *p* = 0.773), nor a condition by mark interaction (*F*_(*4, 60*)_ = 0.437, *p* = 0.782). There was no effect of trial (*F*_(*1, 51*)_ = 0.883, *p* = 0.352), nor a mark by condition by trial interaction (*F*_(*4,51*)_ = 0.436, *p* = 0.782) when this factor was included in the model. An effect of mark colour was also found when examining the number of feather ruffles (*F*_(*2, 60*)_ = 3.503, *p* = 0.036; Fig. S4) as birds made the most ruffles when wearing a red mark. This preference to identify the red mark regardless of viewing condition indicates this subset of birds could either feel the mark or, more likely, glanced the mark in their peripheral vision when preening, consistent with the findings reported for jackdaws^20^.

## Discussion

When the mark test results are considered in conjunction with the results of the caching task, we conclude that the birds were interpreting the blurry image as their reflection and not as another individual. Between both tasks, most nutcrackers showed an advantage for self-recognition when in the presence of a blurry mirror. However, one bird (Fido) may have shown evidence of self-recognition with both the regular and blurry mirrors. During the caching task, Fido did not suppress caching with either mirror type (though there was also no suppression to an actual conspecific ([*M*_*Baseline*_ ± *SEM* = 26.67 ± 3.02; *M*_*Alone*_ ± *SEM* = 23.83 ± 2.96; *M*_*Mirror*_ ± *SEM* = 24.83 ± 3.48; *M*_*Blurry*_ ± *SEM* = 25.17 ± 3.84; *M*_*Observed*_ ± *SEM* = 23.83 ± 1.94]; Table S1), and during the mark test, Fido reliably made actions to the mark region only when the red mark was applied during mirror conditions. Therefore, it seems nutcrackers are capable of self-recognition in the sense that they understood their bodies could be represented in an external medium, although learning their specific features proved much more difficult. The results of Fido however, indicate this ability may not be outside the capabilities of this species, though the learning history of individuals (i.e., cue competition resulting from reliable natural associations between contingent motion and ‘self’ and between identity information and ‘others’) may bias against learning to self-recognize with high quality reflections. It is still unclear why some individuals of a species are able to overcome this prior learning and others are not – a common feature of most mark test studies, where only a small subset of animals pass the test^7^,^8^,^9^,^24^.

Although these results may be interpreted as support for self-recognition, it is also possible that the focal birds did not appreciate that the blurred image was a reflection during the caching task. This may have occurred either because they interpreted the blurred image as a conspecific that was unable to see them well enough to pose a risk (as they were unable to clearly see the individual), or the focal bird was unable to recognize the blurred image as a bird at all. However, it is unlikely the birds’ understanding of the blurred image changed so fundamentally between the caching experiments and the mark test, the latter of which strongly suggests the birds responded to the blurred image as their reflection. Further testing will certainly be required to confirm this preliminary finding. In particular, an additional caching condition, during which a focal bird caches with an observer placed behind a ‘blurred’ acrylic barrier, would be useful for validating the mirror-caching task as a stand-alone measure of self-recognition.

The blurry mirror resembles conditions the birds experience naturally: distorted reflections, such as in water; and self-generated visual information only containing contingent motion information, such as a shadow. On exposure to a regular mirror, the less familiar identity information may interfere with the birds’ ability to attend to the more diagnostic contingent motion information required to foster self-recognition. The blurry mirror likely acted as a band-pass filter removing the high spatial frequencies associated with identity information, allowing the birds to attend to contingent motion information through global cues provided by low spatial frequencies^26^. Developmental evidence from children has also indicated contingent motion is particularly important for self-recognition^27^. The dissociated use of identity and contingent motion information discovered here undermines previous negative findings with other species examined for mirror use, particularly corvids^20^,^28^,^29^.

Our work advances the study of self-recognition in three important aspects. First, we deviate from the predominant approach of relying solely on the mark test by supplementing the mark test with a task exploiting an ecologically relevant behaviour (i.e., caching). Using ecologically relevant behaviours has the advantage of more easily tapping into a species’ natural cognitive abilities as it ensures the animal engages in behaviour they are motivated to express. Such motivational differences among species for removing marks or interacting with mirrors can interfere with identifying self-recognition^3^,^4^. With our approach the animal is not forced into an interaction with a mirror, rather, self-recognition can emerge as a by-product of the animal’s social or foraging behaviour. Second, this is the first study to show a corvid self-recognizing during the mark test that cannot be accounted for by the use of tactile cues^20^, providing evidence of convergence between the mental abilities of corvids and primates^30^ and that a mammalian neocortex is not necessary for self-recognition^9^. However, the capacity for self-recognition likely varies by species along an evolutionary gradient according to their ecology. Third, using blurred reflections enables us to examine what visual information is important for self-recognition and provides a potential gradient by which we can assess different species.

Our results suggest the larger psychological construct of self-recognition can be broken into component abilities^31^, use of identity and contingent motion information^32^, thereby facilitating more useful comparative evolutionary analyses of this trait^33^. Thus, instead of limiting self-recognition to a binary, pass/fail decision, we can now add nuance by asking what are the component abilities of mirror self-recognition, what components are present in any given species, and how these components progress throughout development in both human and non-human animals.

## Additional Information

**How to cite this article**: Clary, D. and Kelly, D. M. Graded Mirror Self-Recognition by Clark’s Nutcrackers. *Sci. Rep.* **6**, 36459; doi: 10.1038/srep36459 (2016).

**Publisher’s note:** Springer Nature remains neutral with regard to jurisdictional claims in published maps and institutional affiliations.

## Supplementary Material

Supplementary Information

## Figures and Tables

**Figure 1:**
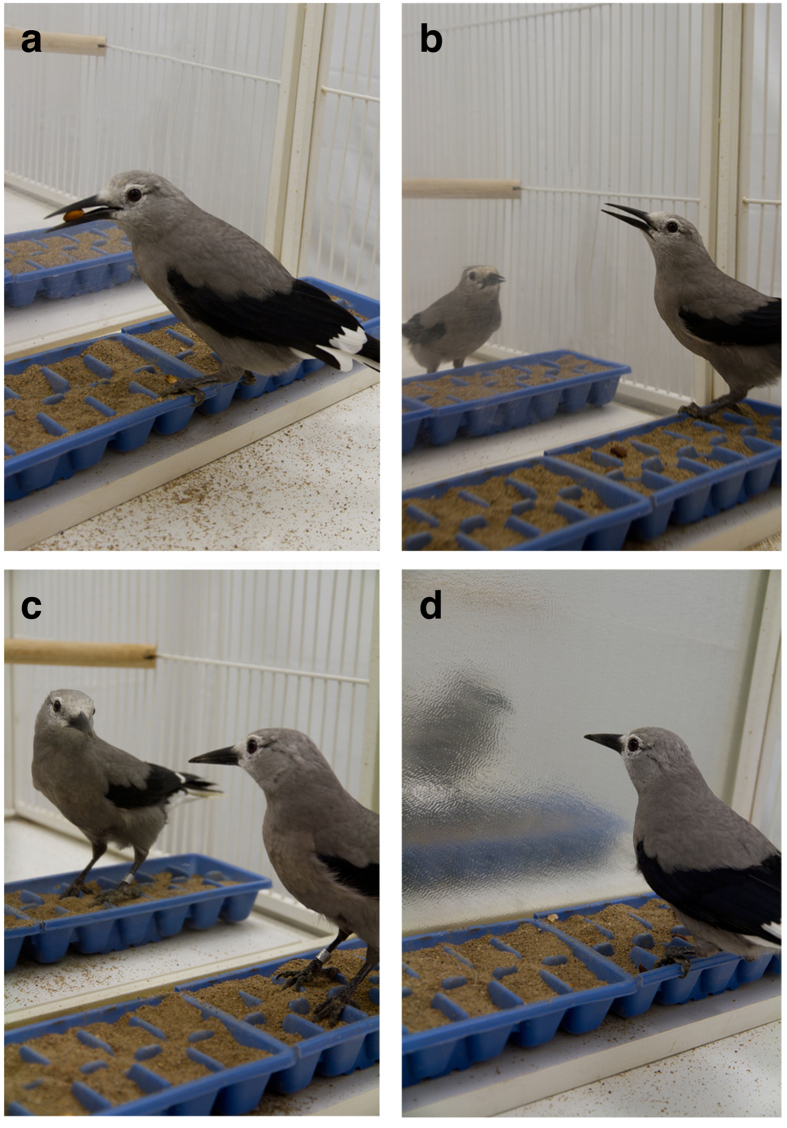
Photos showing the (**a**) alone, (**b**) observed, (**c**) regular mirror, and (**d**) blurry mirror caching conditions of Experiment 1.

**Table 1:** Number of mark actions (non-mark actions) [the sum of trials 1 and 2] during each condition with each mark.

Subject	Barrier/Grey	Barrier/Red	Blurry /Grey	Blurry /Red	Mirror/Grey	Mirror/Red	Regular Mirror	Blurry Mirror	Non-Mirror Visual
Fido	0 (12)	0 (5)	1 (3)	**20** (13)	5 (14)	**64** (37)	☑	☑	
Bitsy	0 (0)	0 (2)	0 (17)	**19** (2)	0 (1)	**2** (3)	☑	☑	
Jan	1 (32)	4 (31)	0 (23)	**19 (**94)	0 (4)	3 (2)		☑	
Reorx	1 (0)	0 (4)	1 (7)	**4** (1)	0 (7)	0 (10)		☑	
Lance	1 (2)	1 (0)	0 (40)	**5** (4)	2 (48)	2 (2)		☑	
Capone	0 (31)	5 (47)	5 (87)	**9** (43)	0 (70)	1 (18)		☑	
Krusty	6 (21)	16 (25)	20 (17)	**27** (39)	14 (82)	18 (32)			☑
Sid	24 (176)	22 (184)	6 (153)	17 (115)	0 (138)	**30** (189)			☑
Stefen	1 (6)	10 (4)	0 (13)	0 (5)	1 (4)	2 (2)			☑
Bert	1 (21)	28 (20)	0 (13)	7 (8)	0 (22)	8 (2)			☑

*Note:* Bold values indicate mark action mirror scores that are higher than any control condition. To emphasize important differences, checkmarks in the Regular and Blurry Mirror columns indicate the bird made 1.5x more mark actions during the mirror condition than during any control condition (the value being selected as a conservative subjective point for comparison that could be scaled to each bird’s behaviour). Checkmarks in the Non-Mirror Visual column indicate mark actions were elevated during the Barrier/Red condition.
