# Supplementary material for: Graded Mirror Self-Recognition by Clark’s Nutcrackers
Source: Sci Rep. 2016 Nov 4;6:36459. doi: 10.1038/srep36459 (PMC5095562; doi:10.1038/srep36459)
Supplement: Supplementary Information [file srep36459-s1.pdf]

Graded Mirror Self-Recognition by Clark's Nutcrackers

Dawson Clary[1] & Debbie M. Kelly[1]

[1]Department of Psychology, University of Manitoba,

Winnipeg, Manitoba, CANADA

Supplementary Table S1. **Mean number of caches (±SEM) of each bird during caching experiment 1**

Subject	Baseline	Alone	Blurry	Observed	Mirror
**Fido**	26.67 ± 3.02	23.83 ± 2.96	25.17 ± 3.84	23.83 ± 1.94	24.83 ± 3.48
**Bitsy**	20.33 ± 2.67	20.00 ± 1.88	27.17 ± 1.85	5.50 ± 3.81	8.50 ± 3.89
**Jan**	18.67 ± 3.62	11.67 ± 1.48	9.83 ± 2.46	12.17 ± 2.06	8.67 ± 1.52
**Reorx**	23.67 ± 3.77	13.67 ± 2.58	9.50 ± 1.34	10.50 ± 1.18	8.83 ± 2.09
**Lance**	30.67 ± 1.69	25.67 ± 2.19	18.50 ± 3.69	13.50 ± 0.85	14.33 ± 1.41
**Capone**	18.83 ± 2.57	17.67 ± 3.11	24.17 ± 1.85	13.83 ± 2.09	6.00 ± 2.89
**Krusty**	35.67 ± 0.76	33.83 ± 2.24	36.00 ± 0.37	28.50 ± 2.05	22.83 ± 4.45
**Sid**	24.83 ± 2.24	9.33 ± 2.78	9.50 ± 2.49	3.83 ± 1.38	0.50 ± 0.22
**Stefen**	8.83 ± 2.04	27.00 ± 3.25	24.50 ± 3.80	19.50 ± 4.74	12.67 ± 4.88
**Bert**	20.50 ± 3.41	16.00 ± 2.53	12.00 ± 3.20	8.83 ± 2.27	8.00 ± 1.88

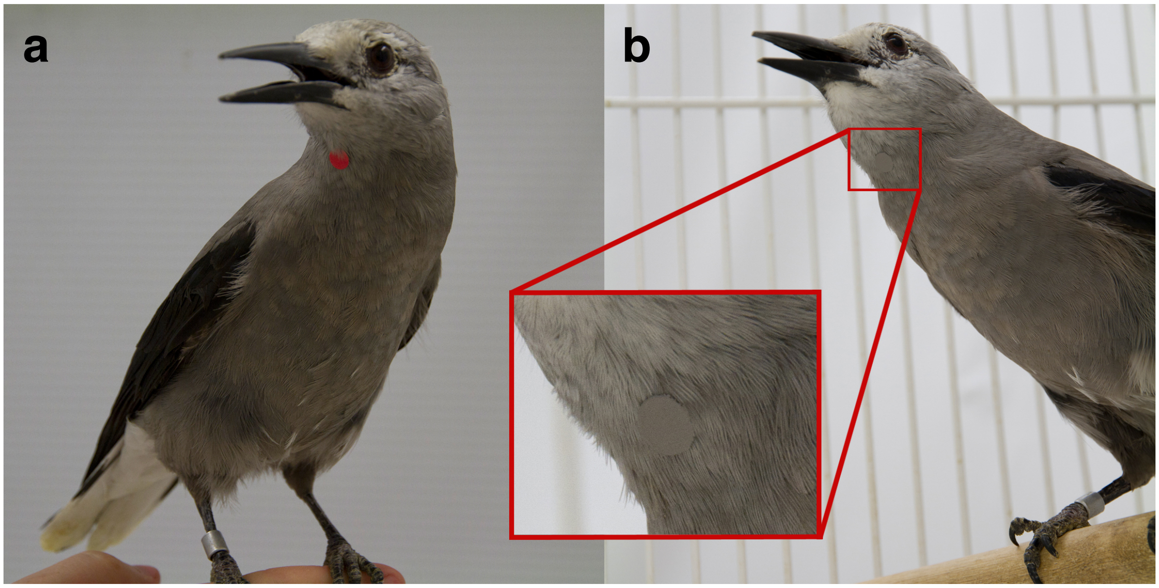

Supplementary Figure S1. Photos showing the placement of a) red and b) grey marks under the bird's beak. Zoomed image b) is shown to highlight the colour match between the grey mark and the bird's plumage.

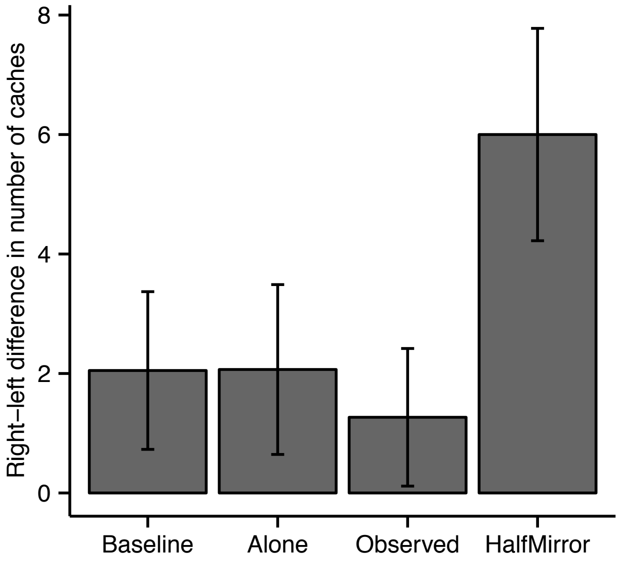

Supplementary Figure S2. Difference between caches made to the non-mirror (right side of tray) and mirror (left side of tray) sides of the tray (±SEM) for all birds (n = 10). A stronger, though non-significant, preference to cache on the non-mirror side, as shown by a larger difference score, was found during the half mirror trials (*M*±*SEM* = 6.00±1.78) relative to the baseline (*M*±*SEM* = 2.05±1.32, z = 2.195, p = 0.091), alone (*M*±*SEM* = 2.07±1.42, z = 1.893, p = 0.177) and observed (*M*±*SEM* = 1.27±1.15, z = 2.278, p = 0.075) conditions suggesting the birds were caching away from the visual presence of the reflection.

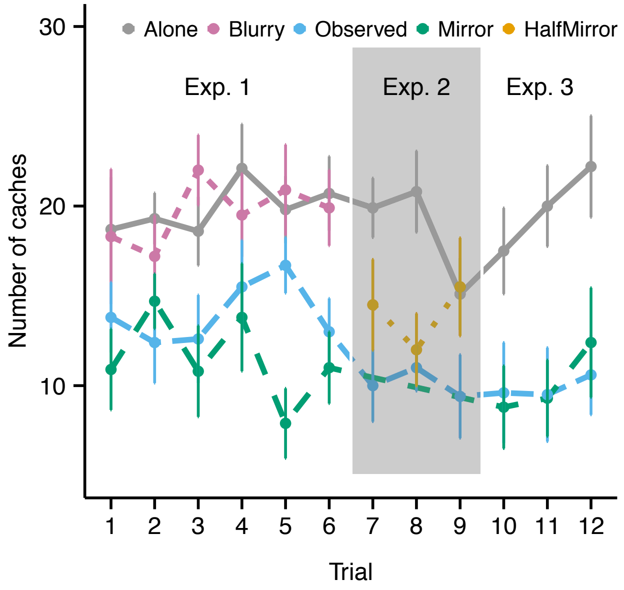

Supplementary Figure S3. Number of caches made (±SEM) by the birds (n = 10) during each condition over the course of all experiments. No effect of trial was found for the number of caches made during any of the conditions ($p > 0.05$).

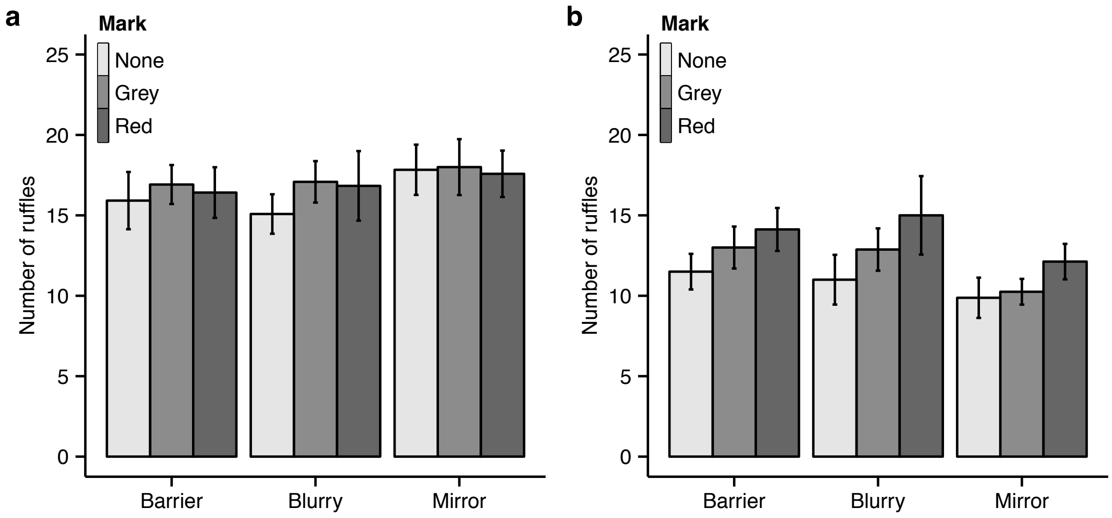

Supplementary Figure S4. Number of feather ruffles (±SEM) across the mirror and mark conditions for a) the six birds showing evidence of mirror use (*Mirror advantage* group), and b) the four birds with no evidence of mirror use (*Non-mirror visual strategy* group). For the *Mirror advantage* group a), no effect of mark ($F_{(2,94)} = 0.365$, $p = 0.695$), condition ($F_{(2,94)} = 0.877$, $p = 0.420$), nor any interaction ($F_{(4,94)} = 0.134$, $p = 0.970$) was found for feather ruffling. For *Non-mirror visual strategy* group b) there was only an effect of mark ($F_{(2,60)} = 3.503$, $p = 0.036$), as these four birds ruffled more when wearing a red mark suggesting mark detection was not overtly mirror-guided, but more visually-based than tactilely-based.
